# Phosphorylated ATF1 at Thr184 promotes metastasis and regulates MMP2 expression in gastric cancer

**DOI:** 10.1186/s12967-022-03361-3

**Published:** 2022-04-09

**Authors:** Tong Li, Huiyuan Cao, Sa Wu, Peimin Zhong, Jie Ding, Jing Wang, Fangfang Wang, Zhiwei He, Guo-Liang Huang

**Affiliations:** 1 Guangdong Provincial Key Laboratory of Medical Molecular Diagnostics, The First Dongguan Affiliated Hospital, Guangdong Medical University, Dongguan, China; 2 Department of Pathology, Chongqing Renji Hospital, University of Chinese Academy of Science, Chongqing, China; 3 Cancer Center, Affiliated Hospital of Guangdong Medical University, Zhanjiang, People’s Republic of China; 4 Laboratory Department, Dongguan Dalang Hospital, Dongguan, China

**Keywords:** Gastric cancer, ATF1, Phosphorylation, MMP2

## Abstract

**Background:**

Studies have revealed an important role of activating transcription factor 1 (ATF1) and phosphorylated ATF1 at Ser63 in tumors. Our previous study identified Thr184 as a novel phosphorylation site of ATF1. However, the role of phosphorylated ATF1 at Thr184 (p-ATF1-T184) in tumor is unclear. This study figured out the role of p-ATF1-T184 in the metastasis of gastric cancer (GC) and in the regulation of Matrix metallopeptidase 2 (MMP2).

**Methods:**

Immunohistochemical analysis (IHC) was performed to analyze the level of p-ATF1-T184 and its relationship with clinicopathological characteristics. Wound scratch test, Transwell assay were used to observe the role of p-ATF1-T184 in the invasion and metastasis of GC. The regulation of MMP2 by p-ATF1-T184 was investigated by a series of experiments including quantitative RT-PCR, western blot, gelatin zymography assay, Chromatin immunoprecipitation (ChIP), luciferase reporter assay and cycloheximide experiment. The Cancer Genome Atlas (TCGA) data were used to analyze the expression and prognostic role of ATF1 and MMP2 in GC. Mass spectrometry (MS) following co-immunoprecipitation (co-IP) assay was performed to identify potential upstream kinases that would phosphorylate ATF1 at Thr184.

**Results:**

High expression level of p-ATF1-T184 was found and significantly associated with lymph node metastasis and poor survival in a GC cohort of 126 patients. P-ATF1-T184 promoted migration and invasion of gastric cancer cells. Phosphorylation of ATF1-T184 could regulate the mRNA, protein expression and extracellular activity of MMP2. P-ATF1-T184 further increased the DNA binding ability, transcription activity, and stabilized the protein expression of ATF1. Moreover, TCGA data and IHC results suggested that the mRNA level of ATF1 and MMP2, and protein level of p-ATF1-T184 and MMP2 could be prognosis markers of GC. Two protein kinase related genes, LRBA and S100A8, were identified to be correlated with the expression ATF1 in GC.

**Conclusion:**

Our results indicated that p-ATF1-T184 promoted metastasis of GC by regulating MMP2.

**Supplementary Information:**

The online version contains supplementary material available at 10.1186/s12967-022-03361-3.

## Introduction

Activating transcription factor 1 (ATF1) is a transcription factor belonging to the ATF/CREB family [1]. ATF1 and CREB regulate transcription of many target genes by way of homo-or heterodimerization within the family or with other b-zip transcription factors, which relevant to an AP1 or cAMP-response element [2]. By regulating the expression of downstream target genes, ATF1 is involved in multiple cellular processes, such as cell survival and proliferation [3, 4]. For example, at the same time of the BALB/C3T3 cells transformation induced by NiCl_2_, ATF1 was induced to be up-regulated and subsequently inhibit the transcriptional expression of thrombospondin 1 (TSP1) [5]. In PC12D cells, ATF1 with dominant negative mutation can block the cyclic AMP-induced neuronal axis growth [6]. Su et al. reported the overexpression of ATF1 in nasopharyngeal carcinoma, which was positively correlated with the expression of MMP2 and other genes [7]. ATF1 is overexpressed in esophageal squamous cell carcinoma and positively correlated with lymph node metastasis and early tumor invasion [8].

Many studies have shown that the activity of transcription factors is usually regulated by multiple phosphorylation sites. These sites modulate the ability of DNA binding, protein stability, and interaction with other transcription factors, thereby regulating the transcriptional activity of transcription factors [9, 10]. Phosphorylated ATF1 at Ser63 has been recognized as an important site for its transcription activity. CDK3 phosphorylates ATF1 at Ser63 and enhances the transactivation and transcriptional activity of ATF1 [11]. Our previous study identified Thr184 as a novel phosphorylation site of ATF1. The transcriptional activity of ATF1 was found to be regulated by Thr184 phosphorylation in nasopharyngeal carcinoma [12]. However, the role of phosphorylated ATF1 at Thr184 in tumor is still unclear.

Our work found out a high level of phosphorylated ATF1 at Thr184 (p-ATF1-T184) in gastric cancer (GC) through a screen of multiple tumors. Gastric cancer (GC), with its incidence rate ranked fifth of cancer, is the third leading cause of death from cancer [13]. Most GC patients have lymph node metastasis at the time of initial diagnosis or surgical resection, which leads to poor prognosis [14]. Thus, the study of the metastasis of GC is of great significance for improving the therapeutic effect and survival rate of GC. In this study, the biological function and clinical significance of p-ATF1-T184 in GC were identified. Our data further showed that p-ATF1-T184 promoted metastasis of GC by regulating the activity of Matrix Metalloproteinase 2 (MMP2).

## Materials and methods

### Antibodies and reagents

Antibodies against ATF1 and MMP2 were from Abcam, Inc (Cambridge, MA, USA). Antibody against phosphorylated ATF1 (Thr184) was raised in a rabbit and was tested in our previous study [12].

### TCGA data, TMA, patients and IHC analysis

TCGA data of gastric cancer with the mRNA expression of ATF1 and MMP2 were downloaded from TCGA (https://www.cancer.gov/tcga). The cutoff values of ATF1 and MMP2, which were originated from TCGA, were optimal for survival analysis [15].

Digestive system tumors tissue microarrays (TMAs), containing 60 cases,10 cases of gastric cancer,10 cases of esophageal cancer,10 cases of colon cancer, 10 cases of colorectal cancer, 10 cases of pancreatic cancer, 10 cases of liver Cancer, were purchased from US Biomax (GI1441, US Biomax Inc). A tissue microarray consisting of 36 pairs of nasopharyngeal carcinoma samples and their adjacent non-tumor tissue samples was also purchased from US Biomax (NPC961. US Biomax Inc). The specimens of 10 cases of glioma were from the Department of Brain Surgery and the 126 gastric cancer specimens obtained in this experiment were from patients received standard radical gastrectomy in the Gastrointestinal Surgery Department of the Affiliated Hospital of Guangdong Medical University during June 2006 to July 2008. No other treatment, such as radiotherapy or chemotherapy, was received preoperatively. The study protocol was approved by the Ethics Committees of Guangdong Medical University. The patients/participants provided their written informed consent to participate in this study.

For all tissues, p-ATF1-T184 and MMP2 expression was detected by IHC. IHC staining was carried out using anti-p-ATF1-T184 and anti-MMP2 antibodies which was performed as previously described [12]. The immunohistochemistry results were scored independently by two individuals using the double-blinded method. The brownish yellow granules that are evident within cells indicate positive staining cells. The IHC scoring method for p-ATF1-T184 staining (mainly nuclear staining) was as follow: the number of positive cells in 5 different fields of the same tissue sample were counted. The mean number of five fields was used as the IHC score [16]. When the primary antibody was MMP2 (mainly cytoplasmic staining), the scoring methods: no staining, < 20% staining: ‘-’. weak staining, 20%-39% staining: ‘ + ’. strong staining, 40%-75% staining: ‘ +  + ’. strong staining, > 75% staining: ‘ +  +  + ’..

### Cell culture

In this study, five gastric cancer cell lines were used: MGC803 (mucinous adenocarcinoma), BGC823, MKN45, SGC7901, AGS (poorly differentiated tubular adenocarcinoma). Immortalized human renal epithelial lines HEK293 were also used in this experiment. All cell lines were authenticated using short tandem repeat (STR) method, performed by Suzhou Jin Wei Zhi Biotechnology Co. Ltd. All gastric cancer cell lines were cultured in RPMI 1640 medium (Gibco, Inc, Shanghai, China) and HEK-293 cell cultured in DMEM medium (Gibco, Inc, Shanghai, China) supplemented with 10% fetal bovine serum (Gibco, Inc, USA), and maintained at 37 °C in a humidified atmosphere with 5% CO_2_.

### Construction of plasmids, small interfering RNAs (siRNAs) and transfection

ATF1-ORF were polymerase chain reaction (PCR)-amplified and cloned into expression vector pcDNA6.0/Myc-His B vector (Invitrogen, Carlsbad, CA). ATF1 mutants Thr184Ala (T184A) and Thr184Asp (T184D) were constructed by kod-plus-mutagenesis Kit (Toyobo Co, Ltd, Osaka, Japan). The plasmids were verified by agarose gel electrophoresis and DNA sequencing. MMP2 siRNA and control siRNA were purchased from GenePharma (Shanghai, China). The plasmid and siRNA oligo were transfected into gastric cancer cells using jet PEI (Jet PEI, Poly plus-transfection, France) following the manufacturer’s instructions.

### Scratch wound assay

The gastric cancer cell lines MGC803 and BGC823 cells transfected with ATF1-T184 mutant plasmid were counted and seeded on tissue‐culture plastic dishes with 60 mm diameter. After 24 h for transfection, a scratch wound was performed using a sterile pipette tip. Photographs were taken at the same location at 0, 24, and 48 h, 3 visual fields were observed in each cell line.

### Invasion assay

The invasion assay was performed using BD transwell in a 6‐well plate. In brief, 2 × 10^5^ MGC803, BGC823 cells in 500 μl serum‐free medium were seeded on Matrigel‐coated transwell, which were embedded into the medium. At 36 h after invasion, using a cotton swab to carefully wipe off the un-transferred cells and matrix glue (Bedford, MA, USA) in the upper chamber. The cells on the bottom side of the well were stained with Giemsa and counted under a microscope.

### RNA isolation and quantitative RT-PCR

Total RNA was isolated using the TRIzol reagent (Invitrogen, Carlsbad, CA), the cDNAs were prepared from 1 μg of total RNA with the MMLV reverse transcription kit (Promega, Madison, USA), qPCR was performed with Fast Universal SYBR Green Master reagents (Roche, USA) in Thermo 5100 Real Time PCR System (Thermo Fisher Scientific) according to the manufacture’s protocol. The reaction mix had a total volume of 15 μl containing 1 × SYBR Green Master Mix, 5 pmol of forward and reverse primers, and 0.5 μl of cDNA. The reactions were incubated in a 96-well plate at 94 °C for 10 min, followed by 40 cycles of 94 °C for 40 s and 60 °C for 50 s. The primers used for MMP2, ATF1, GAPDH were listed as follow: MMP2-F, 5’-CCT GCA AGT TTC CAT TCC GC-3’. MMP2-R, 5’- CTT CTT GTC GCG GTC GTA GT -3’. ATF1-F, 5’-CAA CTA TTC TTC AGT ATG CAC AGA CC-3’. ATF1-R, 5’-GTT TGC ATA TCT CCT GAT GCA GTT-3’. GAPDH-F, 5’-CTC CTC CTG TTC GAC AGT CAG C-3’. GAPDH-R, 5’-CCC AAT ACG ACC AAA TCC GTT-3’ (Bioengineering Biotechnology (Shanghai) Co. Ltd. China).

### Western blot analysis

Total protein was extracted using IP lysis buffer and quantified with a BCA kit (Thermo Scientific, USA). Whole-cell lysates were fractionated by a polyacrylamide gel, and then transferred to a polyvinylidene difluoride (PVDF) membrane (Millipore, Billerica, MA, USA). Then, the membrane was probed with the primary antibodies overnight at 4℃ and incubated with secondary antibodies 1 h at room temperature the next day. These proteins were visualized by Odyssey Infrared Imaging System (LI-COR Biotechnology, Lincoln, NE, USA).

### Gelatin Zymography Assay

MGC803 cells were spread into a six-well plate and transfected with pcDNA6.0-his/Myc, ATF1-WT, ATF1-T184D and ATF1-T184A plasmids respectively. 5.0 × 10^5^ cells were placed in a 6-well plate, respectively. After different cells were completely adherent to the wall, 1 ml serum-free medium was replaced, and starvation culture was conducted for 24 h. The extracellular medium of the treated cells was absorbed and centrifuged to obtain the supernatant. After incubation in a warm box and staining in Coomassie bright blue, they were eluted to the bright band again and photographed with the gel imaging system.

### Chromatin immunoprecipitation, ChIP

Pierce Agarose ChIP Kit (Thermo. USA) was used to perform ChIP assays. HEK293 cells in good growth condition were placed in 10 cm dishes with 1 × 10^6^ cells in each dish, and transfected with Vector, ATF1-WT, ATF1-T184D and ATF1-T184A plasmids respectively. After transfection for 36 h, Thermo Pierce Agarose ChIP Kit was used for the experiment, according to the instructions.

### Cycloheximide treatment

50 μg/ml cycloheximide was applied to the MGC803 cells transfected with Vector, ATF1-WT, ATF1-T184D and ATF1-T184A plasmids, respectively. The protein was collected at three time points of 0 h, 4 h and 8 h, and the changes of ATF1 were analyzed by western blot.

### Reporter gene assay

Fragments of the human MMP2 promoter (and mutants) were synthesized and subcloned into the pGL3 Basic by Jierui Biotech (Shanghai, China). The reporter genes designated as pGL3-MMP2-WT, pGL3-MMP2-Mut. Cells were co-transfected with MMP2 reporter gene and ATF1 expression plasmids. The MMP-2 promoter luciferase reporter (pGL3-MMP2-WT) construct containing one copy of the human MMP-2 promoter gene region (nucleotides -527 to -26, a possible ATF1 binding site GACGTCA in this region) [17],the pGL3-MMP2-Mut mutated them into TAAGACT. The pRL-TK vector-expressing (Promega) Renilla luciferase was co-transfected to normalize the transfection efficiency. A luciferase assay kit (Promega, Madison, WI) was used according to the manufacturer's protocol.

### Co‐immunoprecipitation (Co‐IP) and mass spectrometry protein identification

Co-IP was performed using MGC803 cells transfected with ATF1-WT or ATF1-T184 plasmids. Cell lysate were incubated with ATF1 antibody overnight at 4 °C. Antigen/antibody complex was bound to Protein A/G magnetic beads for 1 h at RT. Beads were washed twice with IP Lysis/Wash Buffer and once with purified water. The antigen/antibody complex was eluted. The detailed procedure refers to technical guidelines provided by Pierce Classic Magnetic IP/Co-IP Kit (Thermo Fischer Scientific, USA). The protein was separated by SDS-PAGE. Protein bands were revealed by silver staining. Samples of co-immunoprecipitation were sent for analyzation using mass spectrometry by Fitgene Biotech Co.LTD (Guangzhou,China).

### Statistical analysis

Survival was analyzed by Kaplan–Meier survival curve. Chi-square test was used to analyze the relationship between the phosphorylation level of ATF1-T184 and clinicopathological data. Cox regression model was used for single-factor and multi-factor analysis. SPSS 16.0 statistical software package (SPSS, Chicago, IL, USA), and Graph-PAD PRISM 5 (Graph Pad Software, La Jolla, CA, USA) were used to analyze experimental data. P < 0.05 was considered to be statistically significant.

## Results

### High level of p-ATF1-T184 is correlated with the lymph node metastasis and poor survival in gastric cancer

**Fig. 1:**
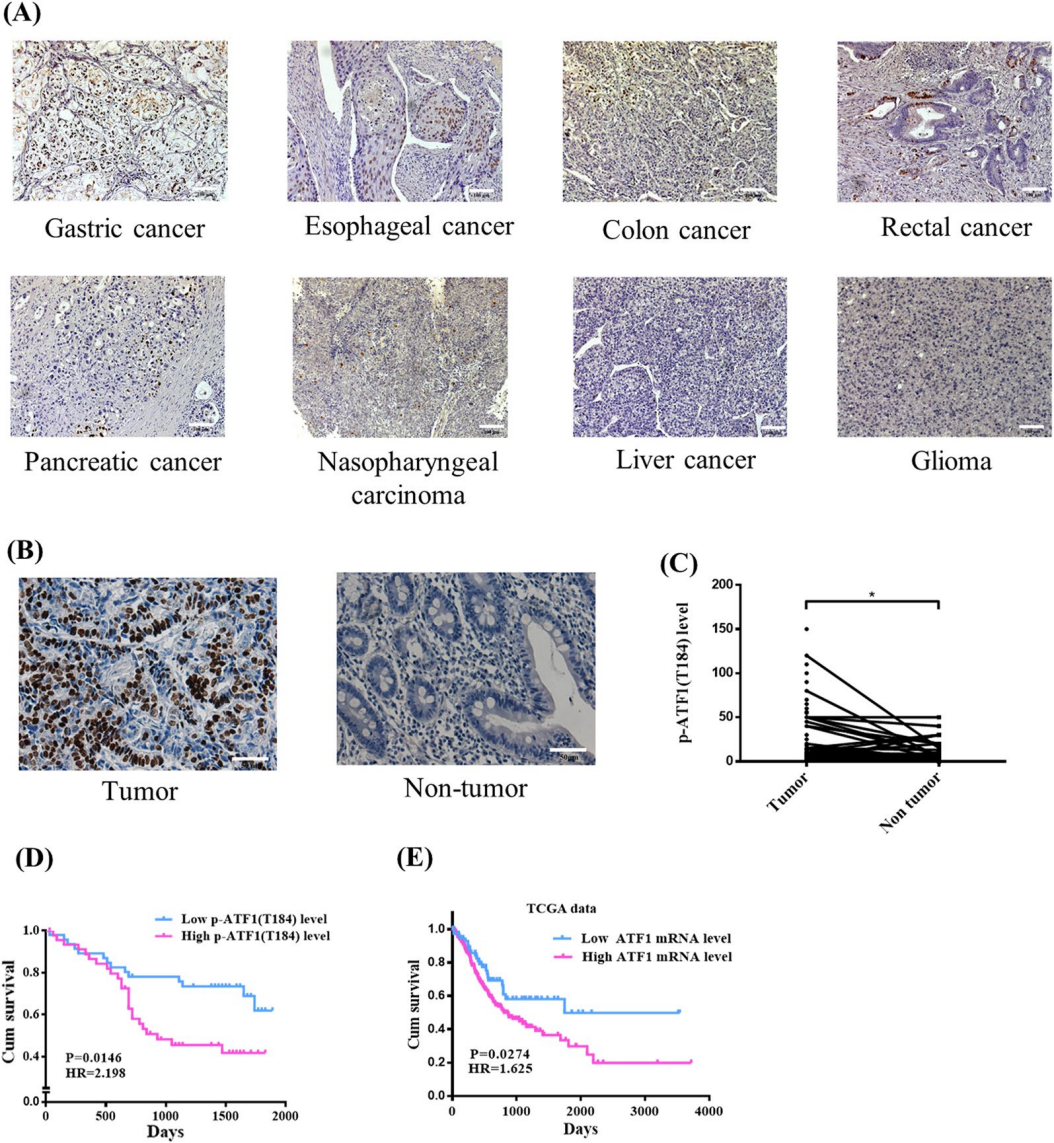
High level of phosphorylated ATF1 at Thr184 is correlated with poor survival in gastric cancer. **A** Immunohistochemical analysis of p-ATF1-T184 in multi-tumor tissues (× 200). **B** GC tissues and non-tumor tissue samples were immunostained for p-ATF1-T184. **C** Level of p-ATF1-T184 in gastric cancer tissue and non-tumor tissue (*P < 0.05). The numbers on y-axis represent the positive staining score of p-ATF1-T184. **D** Kaplan–Meier survival analysis of GC patients according to level of p-ATF1-T184. **E** Kaplan–Meier survival analysis of GC patients according to the mRNA level of ATF1 using TCGA data

The level of phosphorylated ATF1 at Thr184 (p-ATF1-T184) was screened using immunohistochemical analysis in a panel of tumors, including a multi-cancer tissue microarray which included 6 types of digestive system tumors (10 samples per tumor type), a tissue microarray consisting of 36 pairs of nasopharyngeal carcinoma samples and their adjacent non-tumor tissues, and histological tissue sections of glioma. The results showed high levels of p-ATF1-T184 in gastric cancer (5/10) and esophageal cancer (6/10). Moderate expression of p-ATF1-T184 was found in several types of cancer, including colon cancer (4/10), rectal cancer (3/10), pancreatic cancer (3/10), and nasopharyngeal carcinoma (14/36). Immunohistochemistry showed that very few cells were positive for p-ATF1-T184 in liver cancer and glioma (Fig. 1A).

Based on the screening results, our study focused on gastric cancer. The expression of p-ATF1-T184 was investigated in a cohort of gastric cancer with 126 patients using immunohistochemistry. The immunohistochemical results showed that the p-ATF1-T184 expression was significantly higher in tumor tissues than that in non-tumor tissues (Fig. 1B, C). We further analyzed the correlation between p-ATF1-T184 expression and the clinicopathological features of GC patients. As shown in Table1, the p-ATF1-T184 level was significantly associated with lymph node metastasis in patients (P = 0.018). There were no significant correlations between the expression level of p-ATF1-T184 and other characteristics, including age, gender, depth of tumor invasion or histologic type in GC patients (P > 0.05). Then, the Kaplan–Meier method and log-rank test indicated that the overall survival time of GC patients with high p-ATF1-T184 expression was significantly decreased compared with the survival time of patients with low p-ATF1-T184 expression (P = 0.007, Fig. 1D). Univariate and multivariate cox regressions suggested that p-ATF1-T184 expression was a prognosis factor of GC (Table 2). Moreover, the Kaplan–Meier curve showed that a higher mRNA level of ATF1 was associated with poor survival of gastric cancer using TCGA data (Fig. 1E). Collectively, these data showed that high expression of p-ATF1-T184 was associated with the clinical progression and prognosis of GC.

**Table 1:** Correlation between the expression of p-ATF1-T184 and the clinicopathological characteristics of GC patients

Characteristics	P-ATF1-T184		P value
	Low	High	
Age			
> = 50	55	53	0.167
< 50	6	12	
Sex			
Male	43	41	0.378
Female	18	24	
Tumor status			
T1 + T2	7	3	0.145
T3 + T4	51	60	
Lymph node metastasis			
Yes	38	53	0.018*
No	20	10	
Distant metastases			
Yes	8	9	0.938
No	50	54	
Degree of differentiation			
Low	39	36	1.000
High and moderate	13	12	
Pathological stage			
I + II	16	10	0.232
III + IV	29	32	
Family history			
Yes	11	5	0.101
No	38	44	
Tumor size			
> = 5 cm	19	29	0.072
< 5 cm	31	23

**Table 2:** Cox regression analysis of p-ATF1-T184 and MMP2 in gastric cancer with overall survival

Characteristics	HR	CI (95%)	P value
Univariate Cox regression analysis			
P-ATF1-T184	2.399	1.244–4.628	0.009*
MMP2	1.951	1.026–3.708	0.041*
P-ATF1-T184 and MMP2	2.624	1.388–4.959	0.003*
Sex	0.719	0.378–1.367	0.314
Age	0.682	0.266–1.747	0.426
Degree of infiltration	24.011	0.271–2.127 × 103	0.165
Lymph node metastasis	5.018	1.543–16.325	0.007*
Distant metastasis	5.621	2.623–12.046	< 0.001*
Tumor size	1.478	0.750–2.914	0.259
Differentiation	0.617	0.283–1.345	0.225
Multivariate Cox regression analysis			
P-ATF1-T184	1.712	0.872–3.360	0.118
Lymph node metastasis	3.671	1.102–12.225	0.034*
Distant metastasis	3.992	1.843–8.674	< 0.001*
Multivariate Cox regression analysis			
MMP2	1.691	0.868–3.292	0.122
Lymph node metastasis	3.875	1.163–12.787	0.027*
Distant metastasis	4.250	1.955–9.239	< 0.001*
Multivariate Cox regression analysis			
P-ATF1-T184 and MMP2	2.359	1.227–4.533	0.010*
Lymph node metastasis	3.640	1.095–12.095	0.035*
Distant metastasis	4.502	2.066–9.812	< 0.001*

### P-ATF1-T184 affects the migration and invasion of gastric carcinoma cell lines

**Fig. 2:**
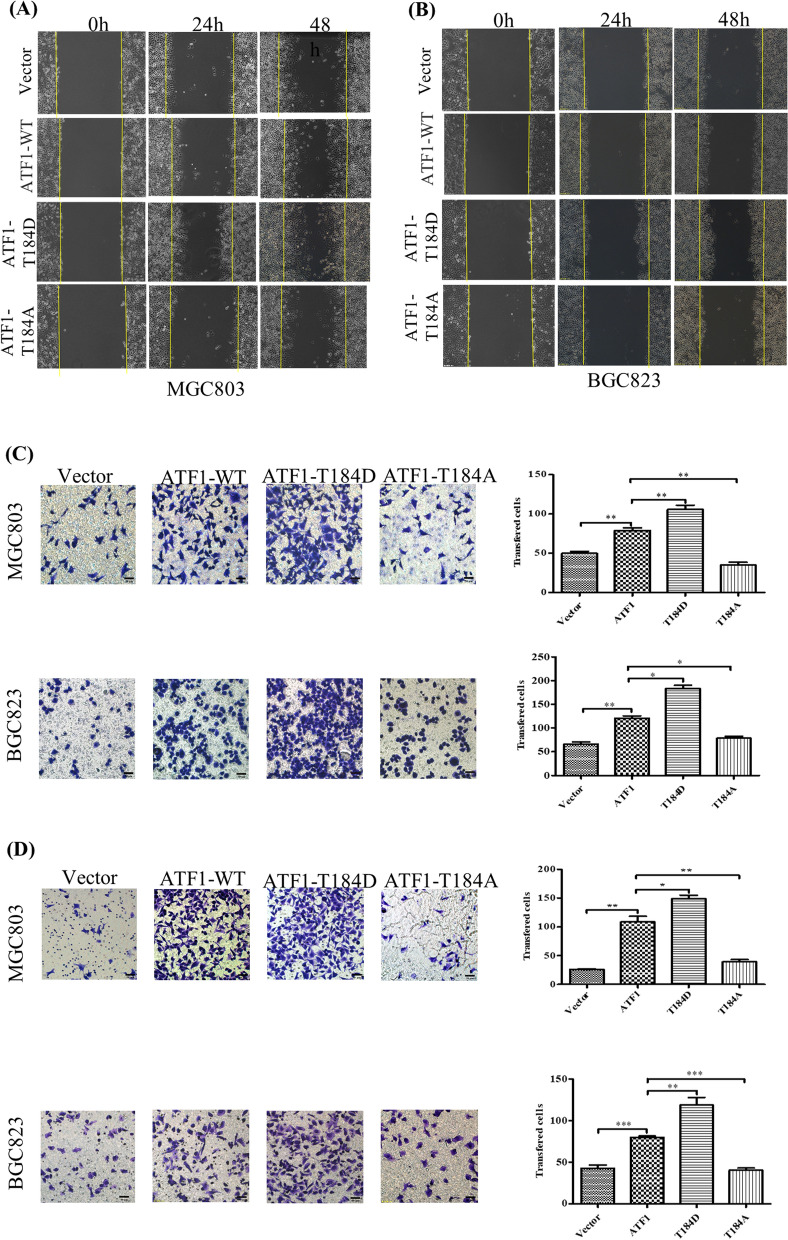
P-ATF1-T184 enhanced the migration and invasion of gastric cancer cells. **A**, **B** Scratch wound assay showing the effect of ATF1- T184 phosphorylation on migration of gastric cancer cells MGC803 and BGC823 (× 100). **C** Transwell assay detected the effect of ATF1-T184 phosphorylation on gastric cancer cell migration (*P < 0.05, **P < 0.01). **D** Matrigel‐coated Transwell assay detected the effect of ATF1-T184 phosphorylation on gastric cancer cell invasion (*P < 0.05, **P < 0.01, ***P < 0.001). Experiments were carried out using transient transfection with corresponding plasmid

Since an association between p-ATF1-T184 level and lymph node metastasis was found, the p-ATF1-T184 influencing migration and invasion of human gastric cancer cells were tested by wound healing assay and transwell assay using gastric cancer cell lines MGC803 and BGC823 transfected with ATF1-T184 wild-type or mutant plasmids. While ATF1-T184D mimicked an active, highly phosphorylated Thr184 of ATF1, ATF1-T184A represented an inactive, dephosphorylated state of Thr184. The wound healing assay showed the gastric cancer cells transfected with the ATF1-T184D mutant plasmids had better migration ability in both cells (Fig. 2A, B). Transwell assay showed that the cell migration and invasion ability were increased in cells transfected with ATF1-T184D mutant plasmids but decreased in cells transfected with ATF1-T184A mutant plasmids (Fig. 2C, D).

### P-ATF1-T184 regulates the expression and activity of MMP2 in GC cells

**Fig. 3:**
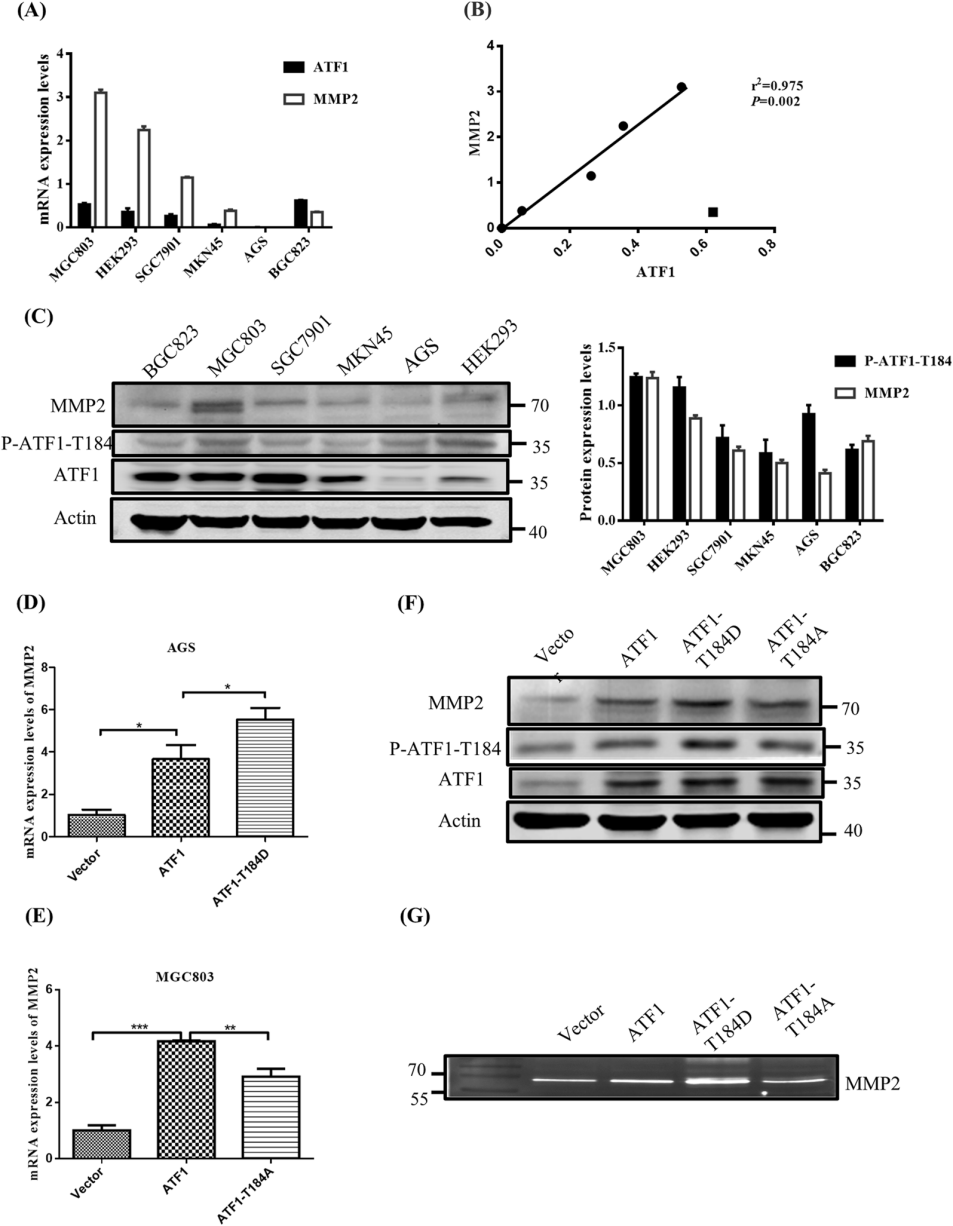
The expression and activity of MMP2 is up-regulated by P-ATF1-T184. A Real-time quantitative RT-PCR detection of ATF1 and MMP2. **B** The correlation analysis between the mRNA expression of ATF1 and MMP2. **C** Western blot showed the expression of phosphorylation ATF1-T184 and protein expression of ATF1, MMP2 in different cell lines. The quantitative data of p-ATF1-T184 protein were presented in the right panel. **D** Effect of ATF1-T184D on mRNA level of MMP2 in AGS cells. **E** Effect of ATF1-T184A on mRNA level of MMP2 in MGC803 cells. **F** Western blot showed the effect of ATF1-T184D and ATF1-T184A on the protein expression of MMP2. **G** Gelatinase assay revealed the effect of ATF1-T184D and ATF1-T184A on the activity of MMP2 in gastric cancer cells MGC803. Experiments were carried out using transient transfection with corresponding plasmid

MMP2 which regulates cell migration and invasion was a transcriptional target of ATF1. This study tried to figure out whether MMP2 was a target of ATF1 that could be regulated by phosphorylated Thr184 in the metastasis of GC. The correlation between ATF1 mRNA and the mRNA levels of MMP2 was examined in gastric cancer cell lines and HEK293 cells. The experimental results showed that MMP2 mRNA expression levels were positively correlated with ATF1 in cancer cells except BGC823 cells (R^2^ = 0.975, P = 0.002, Fig. 3A, B). Western blot analysis confirmed that the protein expression of MMP2 was correlated with the protein level of p-ATF1-T184 except BGC823 and AGS cells (Fig. 3C). Quantitative PCR data showed that MMP2‐mRNA level was increased in AGS cells transfected with ATF1-T184D plasmids, but was significantly decreased in MGC803 transfected with ATF1-T184A, compared to that in cells transfected with ATF1-WT (Fig. 3D, E). The results of western blot further confirmed that the protein expression of MMP2 was higher in MGC803 cells with ATF1-T184D transfection, but lower in cells with ATF1-T184A transfection, when compared to ATF1 wildtype transfection (Fig. 3F). To detect whether MMP2 activities were affected by p-ATF1-T184, a gelatin zymography experiment was performed. As shown Fig. 3G, MMP2 activities in GC cells transfected with ATF1-T184D were higher than those transfected with ATF1 wildtype. MMP2 activities in GC cells transfected with the ATF1-T184A were lower than those transfected with ATF1 wildtype. These data suggested that the expression and activity of MMP2 were up-regulated by p-ATF1-T184.

### P-ATF1-T184 enhances the DNA binding activity of ATF1 and stabilizes its expression

**Fig. 4:**
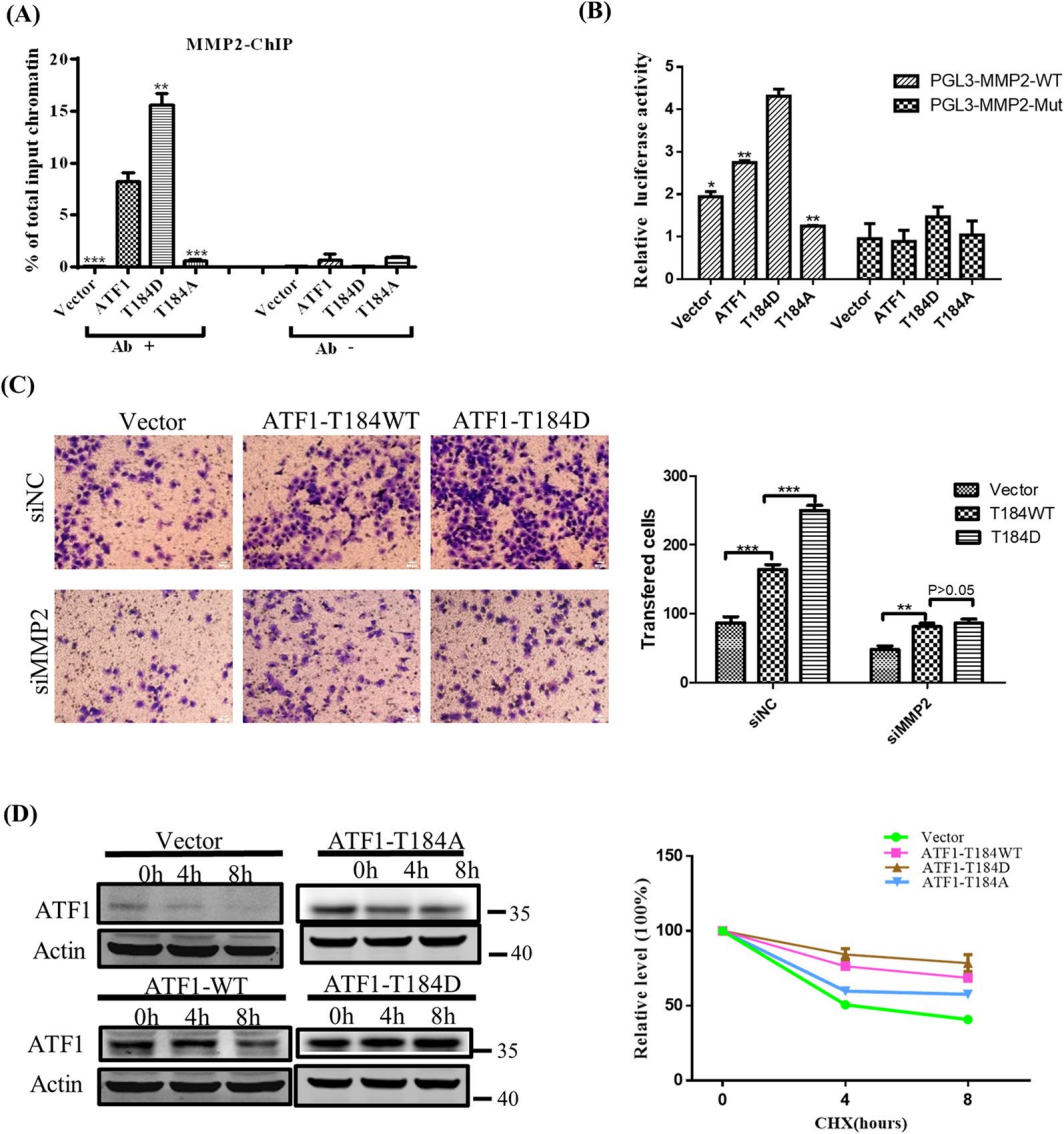
P-ATF1-T184 influences the DNA binding activity and stability of ATF1. **A** ChIP experiment showed the influence of ATF1-T184D and ATF1-T184A on DNA binding ability of MMP2 in HEK293 cells. **B** Luciferase reporter assays showed p-ATF1-T184 promoted the activity of MMP2 promoter. **C** The invasion capabilities of p-ATF1-T184 were impaired by MMP2 knockdown in BGC823 cells. **D** Cycloheximide experiment showed effect of p-ATF1-T184 on the stability of ATF1 protein in MGC803

To investigate the influence of p-ATF1-T184 on the DNA binding activity to MMP2, the chromatin-immunoprecipitation (ChIP) assay was performed using HEK-293 cell lines. Our result showed ATF1 DNA binding ability to MMP2 was higher in cells transfected with ATF1-T184D, but lower in cells transfected with ATF1-T184A, when compared to ATF1 wildtype transfection (Fig. 4A). To further demonstrate the regulation by p-ATF1-T184 on MMP2, luciferase reporter assays were performed. Luciferase vectors containing either wild type or mutant promoters of MMP2 were co-transfected with ATF1-T184 wild-type or mutant plasmids in HEK-293 cells. The results indicated that ATF1-T184D enhanced the luciferase activity of the MMP2-WT compared to ATF1-WT; but insignificant enhancement was found in that of the MMP2-mut group (Fig. 4B). These results showed that p-ATF1-T184 can promote the transcriptional activity of the MMP2 promoter.

Additionally, we tested whether p-ATF1-T184 affects GC cell invasiveness through MMP2. BGC823 cells were co-transfected with ATF1 plasmids, and siRNA of MMP2. Figure 4C showed cells transfected with ATF1-T184D mutant plasmids were significantly increased compared to that transfected with ATF1-WT in the siRNA control group, while the increasement was impaired by siMMP2(Fig. 4C). These data illustrated p-ATF1-T184 promoted metastasis by regulating MMP2 expression.

We measured the ATF1 protein attenuation by western blotting in MGC803 cells transfected with ATF1 wild-type or mutant plasmids after treatment with cycloheximide (CHX). No significant change of ATF1 protein level was found post CHX treatment in cells transfected with ATF1-T184D. However, significant decay of ATF1 was shown in ATF1-T184A transfected cells post CHX treatment. These findings suggest that p-ATF1-T184 stabilized the protein expression of ATF1 (Fig. 4D).

### MMP2 is a prognosis factor of GC patients

**Fig. 5:**
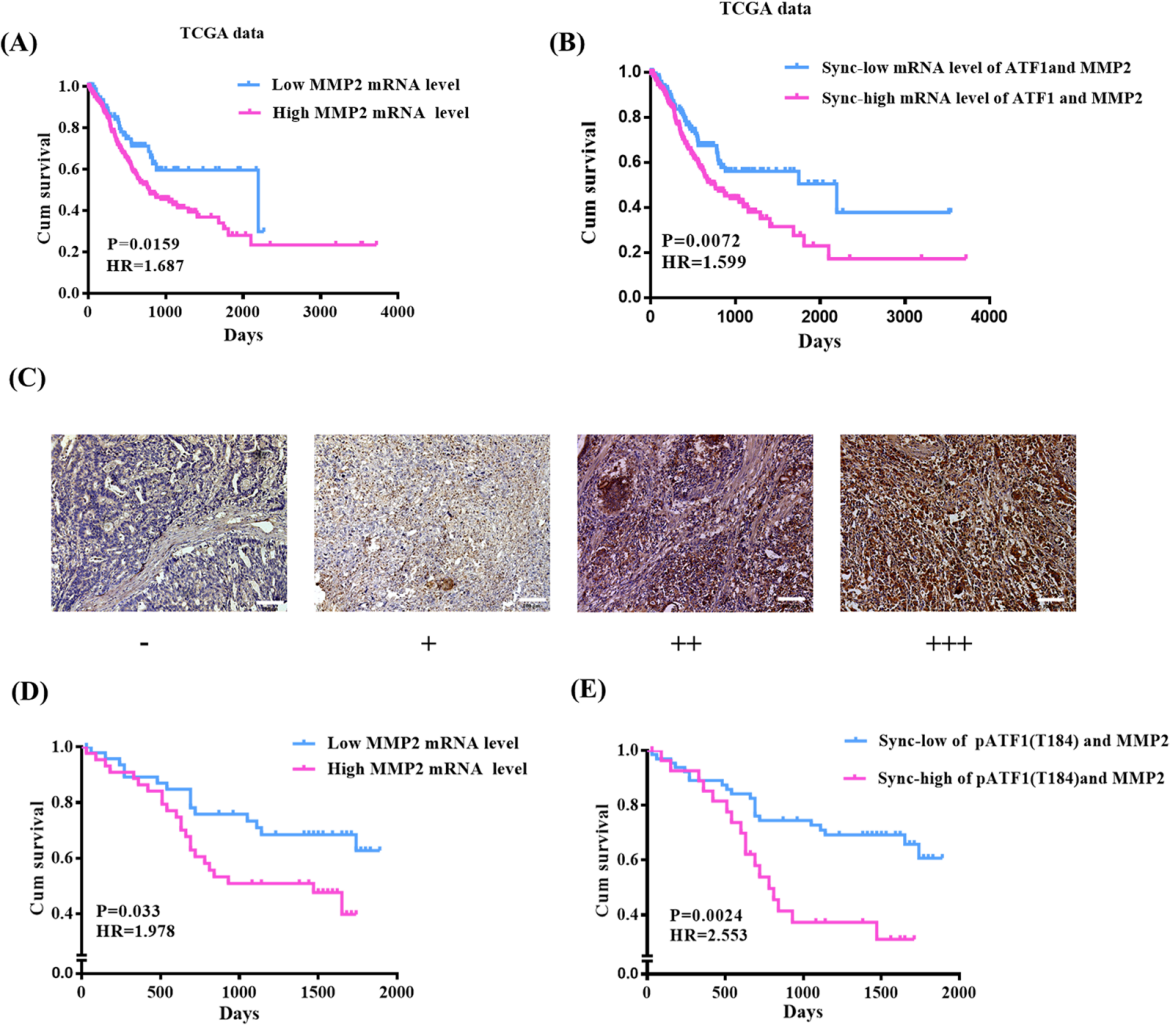
MMP2 is correlated with survival GC patients. **A** Kaplan–Meier survival analysis of GC patients according to the mRNA level of MMP2 using TCGA data. **B** Kaplan–Meier survival analysis of GC patients according to the combined mRNA level of MMP2 and ATF1 using TCGA data. **C** Expression of MMP2 in gastric cancer tissues. **D** Kaplan–Meier survival analysis showed the relationship between the mRNA expression level of MMP2 in gastric cancer tissue and prognosis of patients. **E** Kaplan–Meier survival analysis showed the relationship between the synchronous expression levels of p-ATF1-T184 and MMP2 in gastric cancer tissue and prognosis of patients

With the utility of TCGA data, we found that higher mRNA level of MMP2 was associated with poor survival of gastric cancer by Kaplan–Meier curve (Fig. 5A). Combined mRNA expression of MMP2 and ATF1 was associated with gastric cancer survival (Fig. 5B). Further analysis of MMP2 protein expression in a cohort of gastric cancer was performed using IHC. MMP2 staining in the gastric cancer was scored on a scale of negative (−), weak (+), moderate (+ +), and strong (+ + +) (Fig. 5C). We defined (−) and (+) as low expression, (+ +) and (+ + +) as high expression (Fig. 5C). Then, we used Chi-square test to evaluate the relationship between MMP2 and p-ATF1-T184. As listed in Table3, MMP2 expression was positively correlated with p-ATF1-T184 (P = 0.002). In addition, Kaplan–Meier and log-rank test indicated patients exhibiting high MMP2 expression had shorter survival times than those exhibiting low MMP2 expression (Fig. 5D). As shown in Fig. 5E, patients with concomitantly high expression of p-ATF1-T184 and MMP2 have shorter overall survival than patients with concomitantly low expression. We further examined the relation of the combination of MMP2 and p-ATF1-T184 expression status with the patients’ clinical outcome using the univariate and multivariate Cox regressions (Table2). According to the analysis, high expression of p-ATF1-T184 and high expression of MMP2 were prognosis factors of GC in univariate Cox regression (P = 0.009 and P = 0.041, respectively, Table2). Synchronous high expression of both p-ATF1-T184 and MMP2, lymph node metastasis and distant metastasis were prognosis factors of GC in univariate and multivariate Cox regressions (P = 0.010, P = 0.035, and P < 0.001, respectively, Table 2).

**Table 3:** Correlation analysis of expression of MMP2 and p-ATF1-T184 in gastric cancer

		P-ATF1-T184		
		Low	High	P value
MMP2	Low	41 (32.54%)	26 (20.63%)	0.002
	High	20 (15.87%)	39 (30.95%)	

### The potential kinase regulating phosphorylated ATF1 at Thr184

**Fig. 6:**
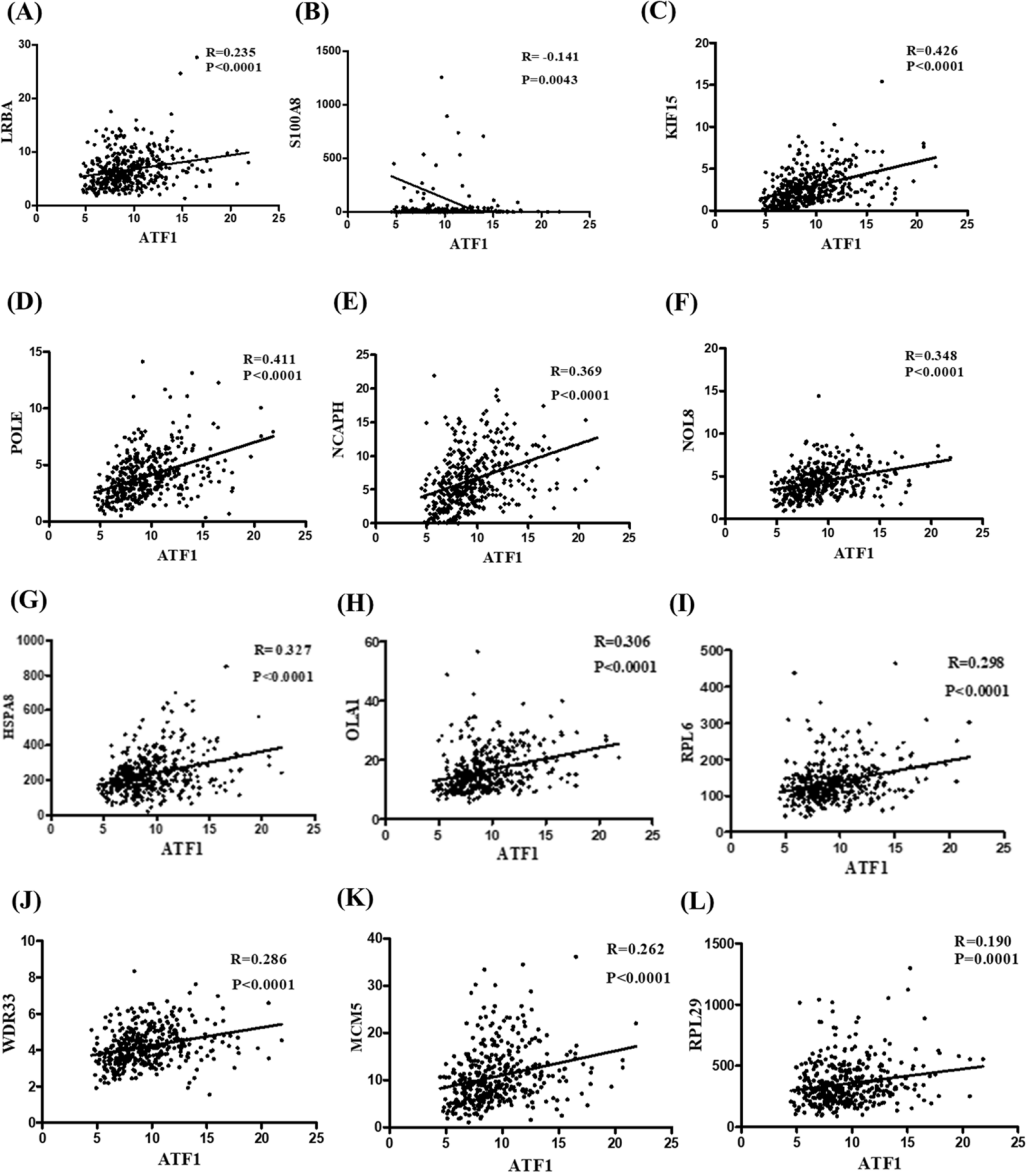
The protein interact with P-ATF1-T184. **A**, **B** LRBA and S100A8 (8 data points are out outside *the* y axis limits for better visualization) protein kinase related genes were correlated with ATF1 in GC. **C**–**L** 8 genes expression displayed weak correlation with the expression of ATF1 (P ≤ 0.0001)

To identify potential upstream kinases that would phosphorylate ATF1 at Thr184, we performed mass spectrometry (MS) following co-immunoprecipitation (co-IP) assays using GC cells transfected with ATF1-WT or ATF1-T184D plasmids. A total of 42 proteins were identified in the immunoprecipitate from GC cells transfected with ATF1-T184D compared to that transfected with ATF1-WT (Additional file 1: Table S1). Correlation analyses using TCGA data indicated that 23 of the 42 genes were correlated with the expression of ATF1 in GC (Fig. 6). Two of the protein kinase related genes, LRBA and S100A8 were found to be correlated with ATF1 in GC.

## Discussion

Transcription factors are widely found in various tissues and cells of the body, recognizing specific DNA sequences to control transcription [18]. ATF1, a member of the activating transcription factor, contains a basic-leucine zipper (b-zip) motif for DNA binding and dimerization [19]. ATF1 regulates cell survival and proliferation by acting either as a transcriptional activator or a repressor [20]. ATF1 is closely related to tumorigenesis and development. ATF1 was first associated with tumors in soft tissue malignant melanoma (clear cell sarcoma). In this tumor, chromosomal translocation T (12;22) (q13; Q12) and the EWS/ATF1 fusion gene are present in the translocation chromosome, which have the nature of oncogene [1]. As a non-fusion protein molecule, ATF1 also plays an important role in tumor development. ATF1 expression was increased in lymphoma cells and activated lymphocytes, suggesting that ATF1 may play an important role in cell growth and differentiation [21]. In human epidermoid tumor cells, dominant negative mutation ATF1 reduces the transcription expression of H-2DD gene [22]. FRA-1 is highly expressed in a variety of tumors and promotes tumorigenesis, while ATF1 can bind to its promoter and play a transcriptional activation role [23].

ATF1 is highly expressed in many types of tumors as a transcription factor. ATF1 overexpression and hyperphosphorylation are associated with the clinical stage of nasopharyngeal carcinoma [7]. Increased ATF1 expression was also found in patients with clear cell sarcomas in the gastrointestinal tract [24], T-cell lymphoma [21], cervical cancer, and thyroid cancer [25]. In this study, we found that the mRNA level of ATF1 was higher in gastric cancer and associated with patient’s survival using TCGA data. Higher level of p-ATF1-T184 was further found in a cohort of gastric cancer and was associated with lymph node metastasis and lower survival rate.

Transcription factor activity is usually regulated by multiple phosphorylation sites, for example, transcription factor RUNX1 is considered to be a major regulator of hemopoiesis and plays an important role in both hematopoietic system tumors and solid tumors. There are multiple functional phosphorylation sites for serine/threonine and tyrosine on RUNX1, phosphorylation of Ser249 and Ser266 promotes the disjunction of RUNX1 from the transcriptional repressor Sin3A, thereby enhancing its transcriptional activity [26]. Another member of the ATF/CREB family of transcription factors, CREB has multiple phosphorylation sites that regulate its transcriptional activity, including Thr100, Ser111, Ser114, Ser117, Ser121, Ser129, Ser133, and Ser142. Among them, Ser133 is an important regulatory site, where point mutations can lead to the inactivation of CREB protein [27]. ATF1 phosphorylation at Ser63 is a major regulatory site which recruits associated coactivators to increase transcriptional activity. PKA and several other serine threonine kinases phosphorylate ATF1 at site Ser63 [11]. In addition, HIPK2 phosphorylates Ser198 but not Ser63. ATF1 phosphorylation by HIPK2 activated ATF1 transcription function, HIPK2 counteracts ATF1 inhibition of ferritin H via ARE [20]. In previous work, we identified ATF1 Thr184 as its new phosphorylation site, which mediates the interaction between ATF1 and Pin1, enhances ATF1 transcription activity and the development of nasopharyngeal carcinoma [12]. In this study, our data indicated that phosphorylated ATF1 at Thr184 enhanced the protein stability and transcription activity of ATF1 in GC.

To identify potential upstream kinase that could phosphorylate ATF1 at Thr184, co-immunoprecipitation assays, mass spectrometry and correlation analysis using TCGA data were carried out. These results indicated that two kinase related genes, LRBA and S100A8 might be involved in the regulation of phosphorylating ATF1 at Thr184. LRBA is found to be overexpressed in several different types of cancers and could be involved in cell proliferation and apoptosis [28].

Like its orthologue AKAP550 (protein A kinase anchor protein) and its isoform neurobeachin, LRBA is suggested to contain protein kinase A (PKA) binding sites and could be a portion of the signaling pathway that needs its interaction with inositol phospholipids and/or PKA [28–30]. LRBA is found to be induced by CD40L and IL-4 stimulation in B cells and be interacted with RIIα and RIIβ, which are two regulatory subunits of PKA. These data suggest that LRBA acts as an A-kinase anchor protein [31]. High level of S100A8 and S100A9 is found to be correlated with enhanced cellular motility and migration through biomembranes in several types of cell systems [32]. It is suggested that S100A8 and S100A9 can activate p38 mitogen-activated protein kinase (MAPK)/nuclear factor kappa B in inflammation and gastric cancer cells invasion [33, 34]. PKA and MAPK are well-known Ser/Thr kinases [35, 36]. We infer that phosphorylating ATF1 at Thr184 could be induced by PKA which mediated by LRBA, or by MAPK which mediated by S100A8. ATF1 mediates various cellular processes by regulating transcription of target genes [2]. ATF1 regulates the down-regulation of TSP-1, leading to increased aggressiveness of thyroid cancer cells [37]. Overexpression of ATF1 in nasopharyngeal carcinoma was positively correlated with the expression of MMP2. ATF1 led to the increasing level of MMP2 expression as well as promotion in lung cancer cell migration and invasion [38]. However, previous study did not show which phosphorylated site of ATF1 was important for the regulation. Our data showed that p-ATF1-T184 promoted the migration and invasion of gastric carcinoma cells. Moreover, the regulation of MMP2 by p-ATF1-T184 was investigated by mRNA expression, protein expression, ChIP analyses and luciferase reporter assays. These results suggested that p-ATF1-T184 could mainly affect the transcription and expression of MMP2 by affecting the DNA binding ability and protein stability of ATF1, thus enhancing the invasion and metastasis ability of tumor cells.

MMP2 was gelatinases, also known as type IV collagenase, which is secreted in an inactive proenzyme form. After activation by hydrolysis, MMP2 can degrade gelatin and various proteins in extracellular matrix, which play an important role in the invasion and metastasis of tumors [39]. MMP2 is found to be mainly released by endothelial cells, monocytes, leukocytes, chondrocytes, platelets, osteoblasts, dermal fibroblasts and keratinocytes [40]. It has been reported that MMP2 expression in esophageal cancer is higher than the corresponding esophageal epithelium [41]. In addition, stronger MMP2 expression was found in the front edge of cancer cell invasion, suggesting that MMP2-positive cancer cells have stronger invasive characteristics and are associated with lymph node metastasis [41]. In this study, using TCGA data, we found that higher mRNA level of MMP2 was associated with patient’s survival in gastric cancer. We also found that higher protein level of MMP2 was associated with survival of patient s in our cohort of gastric cancer. Moreover, the expression of p-ATF1-T184 was positively correlated with MMP2 in gastric cancer tissues. The overall survival time was shortened in patients with simultaneous high expression of both p-ATF1-T184 and MMP2. It could be used as an independent risk factor for prognosis of gastric cancer patients.

In conclusion, our study showed that phosphorylated ATF1 at Thr184 could affect mRNA transcription, protein expression levels and extracellular activities of MMP2, by regulating the DNA binding capacity and protein stability of ATF1. The invasion and metastasis ability of the gastric cancer was modulated by p-ATF1-T184. The expression of p-ATF1-T184 and MMP2 could be a prognosis marker of gastric cancer patients. These results indicated that p-ATF1-T184 promoted metastasis of GC by regulating MMP2.

## Supplementary Information

**Additional file 1:** **Table S1**. ATF1 interacting proteins identified by immunoprecipitation and mass spectrometry. **Figure S1**. Western blotting showed Thr184 affects phosphorylation mutually using BGC823 cells transfected with various plasmids.

## Data Availability

Some of the datasets generated and analysis during the current study are available in the TCGA database: https://www.cancer.gov/tcga, the rest can be obtained from corresponding author on reasonable request.

## Abbreviations

ATF1: Activating transcription factor 1
P-ATF1-T184: Phosphorylated ATF1 at Thr184
MMP2: Matrix metallopeptidase 2
GC: Gastric cancer
IHC: Immunohistochemical
Quantitative RT-PCR: Quantitative real time quantitative PCR
ChIP: Chromatin immunoprecipitation
TCGA: The Cancer Genome Atlas
MS: Mass spectrometry
Co-IP: Co-immunoprecipitation
CHX: Cycloheximide
