# Supplementary material for: Phosphorylated ATF1 at Thr184 promotes metastasis and regulates MMP2 expression in gastric cancer
Source: J Transl Med. 2022 Apr 9;20:169. doi: 10.1186/s12967-022-03361-3 (PMC8994398; doi:10.1186/s12967-022-03361-3)

TableS1 ATF1 interacting proteins identified by immunoprecipitation and mass spectrometry

| Protein name |
| --- |
| HIST1H2L, HRNR, HSPA8, S100A8, RPS4Y2, RPS14, ANXA2, FHDC1, RPS26P11, ANKAR,  ATP5A1, PLOD2, FLG2, NCAPH, BEST2, OCRL, PCLO, WDR33, CRYBG3, MCM5, RPL6,  MAP3K9, KIF15, OLA1, LIPT2, C1orf115, OGFOD2, KMT2C, RPL36A, WDR87, HS1BP3,  GABBR1, RPL29, PEX1, NOL8, APEX2, LRBA, PLP1, AP4B1, RPS2, POLE, CUTA. |

FigureS1 Western blotting showed Thr184 affects phosphorylation mutually using BGC823 cells transfected with various plasmids.


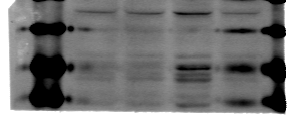

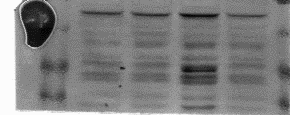


P-ATF1-T184

ATF1

Actin

Vector

ATF1

ATF1-T184D

ATF1-T184A

35

35

40


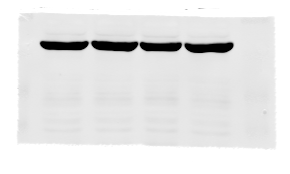

Supplement: Supplementary file 1 — Additional file 1: Table S1. ATF1 interacting proteins identified by immunoprecipitation and mass spectrometry. Figure S1. Western blotting showed Thr184 affects phosphorylation mutually using BGC823 cells transfected with various plasmids. [file 12967_2022_3361_MOESM1_ESM.docx]
